# Organizational happiness and its association with organizational commitment: A mixed-methods study among private school teachers in Türkiye

**DOI:** 10.1371/journal.pone.0348240

**Published:** 2026-05-04

**Authors:** Sema İncekara, Ahmet Şahin

**Affiliations:** 1 Independent Researcher, Alanya, Antalya, Türkiye; 2 Faculty of Education, Alanya Alaaddin Keykubat University, Antalya, Türkiye; Karamanoglu Mehmetbey University: Karamanoglu Mehmetbey Universitesi, TÜRKIYE

## Abstract

**Background:**

Teaching is demoralizing, challenging, and stressful for many teachers. Greater student achievement requires better teacher performance and happiness. An effective school climate in which teachers feel positive emotions is crucial for employing happy and quality teachers. However, given the pressures of the competitive private education sector on teachers, organizational well-being and commitment in the private school setting in Türkiye remain a research gap. Hence, this study aimed to examine the relationship between organizational happiness and organizational commitment of private school teachers, focusing on the factors affecting teachers’ happiness and commitment.

**Methods:**

We conducted a sequential explanatory mixed-methods study. Data from 299 private school teachers were collected for the quantitative strand, while eight teachers from various private schools participated in the qualitative strand. Multiple linear regression analyses were conducted on the quantitative data to examine the relationship between organizational commitment and happiness. Qualitative data were analyzed using thematic analysis with an inductive approach.

**Results:**

The quantitative results revealed high positive affect, fulfillment, affective commitment, and normative commitment, but moderate negative affect and continuance commitment. Positive affect and fulfillment were significantly and positively related to affective, continuance, and normative commitment, whereas negative affect was negatively related to them. Qualitative interviews supported these findings. Positive emotions and supportive work conditions are likely to enhance commitment; however, negative work-related factors may reduce it. Despite their strong affective and normative commitments, teachers’ intentions to remain in school remain unclear.

**Conclusions:**

The study revealed that enhancing teachers’ happiness and creating environments for fulfillment are likely to enhance organizational commitment, whereas reducing negative workplace emotions is vital. Qualitative insights enrich these results by illustrating the factors influencing happiness, such as positive emotions and supportive work conditions, which enhance commitment. Fostering positive effects and decreasing negative factors are key to increasing teachers’ organizational commitment through enhanced happiness.

## Introduction

Today, competitive education market urges schools to supply high quality education for students and parents. Recruiting and retaining highly qualified teachers is both a challenge and necessity for many school leaders [1]. Teacher happiness and commitment in schools are, that’s why, crucial areas of inquiry in organizational psychology and management. Recent research suggests that teaching can often be demoralizing, challenging, and stressful for many teachers [2–4]. This situation is becoming increasingly widespread worldwide and is leading to a significant number of teachers leaving the profession. Indeed, UNESCO [5] indicates that there will be a greater need for teachers in the future. According to the Global Report on Teachers [5], factors such as low salaries, challenging working conditions, heavy workloads, and low professional status are preventing many prospective candidates worldwide from entering the teaching profession or causing them to leave it. All these issues make it even more important to understand the factors that keep teachers healthy and committed to their work. At this point, the positive emotions of teachers and the creation of an effective workplace are crucial for retaining high-quality teachers, which in turn enhances student achievement [6].

Studies on organizational happiness and commitment have gained increasing prominence in management research, mirroring their growing importance in business environments [7,8]. From an individual perspective, happiness is frequently considered a crucial element of a fulfilling life [9]. From an organizational standpoint, allocating resources to increase employee happiness is gaining prominence and has yielded positive outcomes [10].

Teachers often encounter adverse effects in their schools, such as cynicism [11], anxiety [12], stress [13], overwork [14], and burnout [8]. Although some researchers have examined related aspects, including working conditions and teacher accountability [15], satisfaction and motivation [16], and emotional labor [17], work happiness and organizational commitment in Turkish private schools remain understudied [18]. Despite a growing body of research on organizational happiness and commitment, the context of private schools remains a gap in Türkiye. This gap underscores the need for comprehensive research in this field to better understand and address the challenges faced by private school teachers in Türkiye.

### Conceptual framework

#### Happiness at work.

Optimistic emotions, such as happiness, are pivotal for workers in organizational contexts [8]. Organizational happiness encompasses positive and negative emotions alongside employees’ potential realization and is divided into positive affect, negative affect, and fulfillment [19,20]. Positive emotions include joy, pleasure, satisfaction, enjoyment, happiness, peace, and love. Being happy, excited, joyful, enthusiastic, proud, willing, and peaceful in school reflects teachers’ happiness [21]. Thus, positive affect encompasses pleasant emotions and a positive mood, indicating a high level of happiness [20]. Negative emotions, including sadness, anxiety, worry, restlessness, nervousness, anger, boredom, distress, frustration, and depression, are termed negative affect [20,22,23]. In schools, negative affect causes teachers to feel dissatisfied, uneasy, and nervous, with a sense that their efforts are wasted [22,24]. Fulfillment, defined as the cognitive aspect of organizational happiness, involves personal development, potential realization, and self-reflection [20]. It includes achieving one’s potential, developing key abilities, engaging in skill-expressive activities, overcoming challenges, attaining valuable results, advancing life goals, pursuing enjoyable activities and expressing one’s best qualities [23]. Potential realization allows teachers to enhance their performance by demonstrating their skills [22].

### Organizational commitment

Organizational commitment is vital for competing globally and enhancing organizational performance and effectiveness [25]. It has also attracted researchers’ and practitioners’ attention because of its demonstrated impact on factors (e.g., satisfaction, motivation, engagement, retention, and well-being) that are important to organizations [26]. Highly committed employees are more efficient and take on greater responsibilities [27].

Allen and Meyer [28] define organizational commitment as aligning with organizational values through three dimensions: affective commitment, continuance commitment, and normative commitment. Affective commitment involves employees’ emotional attachment to, identification with, and involvement in their organizations. Continuance commitment is based on the costs of leaving, whereas normative commitment stems from a sense of obligation to stay [28]. Affective commitment increases when employees do not want to leave their friends and are satisfied with their work environment [29]. Continuance commitment arises when employees perceive that leaving is costly [30]. In normative commitment, a mindset of obligation causes employees to remain with organization [31].

### Teachers’ commitment and happiness

Research has shown that specific organizational factors, such as organizational culture, leadership, work environment, and trust, positively affect teachers’ commitment to their schools [32]. Organizational commitment also affects teachers’ job satisfaction [33].

Happy employees make better decisions, align themselves with organizational goals, build effective relationships, and positively impact their organizations [34]. Organizational commitment is a key measure of workplace happiness [35]. Studies have shown a positive correlation between teachers’ work happiness and organizational commitment, thus enhancing teacher commitment [18,36]. Happy teachers also have lower turnover intentions [37]. Bahat and Işık [38] reported a significant relationship between teachers’ organizational happiness and commitment. Similarly, Huang et al. [39] noted that kindergarten teachers’ occupational well-being positively predicts their occupational commitment. Arman and Tan [40] revealed that organizational commitment is positively affected by university staff’s happiness. These findings suggest that teachers’ happiness in private schools may increase their commitment levels.

Furthermore, positive emotions expand our immediate repertoire of thoughts and actions, as proposed by Fredrickson [41] in her Broaden-and-Build Theory of Positive Emotions. During moments of positive emotions, negative emotions such as anxiety, sadness, anger, and hopelessness are also suppressed. Thus, positive emotions function as indicators of development and well-being. Thus, positive emotions such as joy, interest, contentment, and love function as indicators of development or well-being. Furthermore, experiencing positive emotions encourages the discovery of new and creative actions, ideas, and social connections, which, in turn, constitute the individual’s personal resources, ranging from physical and intellectual resources to social and psychological resources [41,42]. In this context, positive emotions and satisfaction, which are components of organizational well-being, are expected to strengthen teachers’ commitment to their schools. Conversely, negative emotions, which reflect unpleasant emotional experiences such as anxiety, stress, anger, hopelessness, and sadness, are expected to weaken teachers’ commitment to their schools. Indeed, different studies on teachers’ happiness and commitment have revealed similar relationships [e.g., 18,36,38,40].

## Current study

Education in state schools in Türkiye is free. However, education in private schools is fee-based. Some parents want to send their children to private schools to receive higher-quality educational services. Private schools in Türkiye strive to attract students to their organizations based on many factors, such as the quality of the education services they offer and their fee policies. This has led to competition in the private education market in Türkiye, as in other markets. According to statistics of the 2024–2025 academic year, the number of students attending private schools in Türkiye accounts for 8.6% of the total number of students in formal education [43]. Private schools in Türkiye must address numerous challenges to remain competitive and ensure their sustainability. Reducing costs, enhancing performance, fostering a positive and creative work climate, and improving product and service quality and productivity are becoming increasingly crucial for these schools. However, private school teachers in Türkiye face significant issues compared to teachers working in public schools. Negative working conditions, job insecurity, concerns about the future, insufficient employee rights, excessive demands and expectations from parents, parental pressure, low pay, wage inequity, curriculum problems, long working hours, increasing workload, and role ambiguity [e.g., 12,44–46] are among the leading problems in Türkiye. In other countries as well, private school teachers may face similar challenges. For example, in England, overly demanding parents, parental pressure, and long working hours are sources of issues for private school teachers [13]. In Pakistan, again, issues such as excessive workload, high stress, lack of support, insufficient salaries, inadequate professional development opportunities, and job quit are observed [e.g., 47,48]. At the same time, for Türkiye, another important systemic problem is the frequent turnover of teachers or job quit which are quite common in private schools. This is seen as one of the problems causing qualitative instability and inadequacy in education [49]. All of this makes studies on organizational happiness and commitment both meaningful and necessary in private schools, where employing and retaining qualified teachers is extremely important for increasing competitive educational quality and ensuring stability.

Teacher happiness in school leads to stronger and healthier communication with their students. At this point, teachers’ organizational commitment and happiness significantly influence their effectiveness and efficiency [50]. The teaching profession is highly challenging and stressful [2], with increasing expectations, a need for continuous self-improvement, and diverse responsibilities, all of which lead to stress and burnout. These issues result in poor performance, increased turnover rates, psychological discomfort, and reduced job satisfaction [51]. Conversely, when teachers are happy, it positively affects society, families, employers, and students [52]. Happy teachers work more efficiently, contribute positively to schools, and experience physical and spiritual satisfaction [21,52]. Psychological well-being is a crucial determinant of teachers’ effectiveness and contributes to students’ success. This is because a more positive emotional state is associated with greater well-being [53]. Given the global challenge of increasing teacher attrition and turnover rates [54], high stress [2], burnout [8], and workload [14], exploring factors that improve teachers’ happiness and, in turn, organizational commitment has become a pivotal topic in educational research. Therefore, ensuring teachers’ happiness in schools is crucial for enhancing their organizational commitment, which, in turn, increases student success and organizational productivity. This study underscores the importance of teachers’ happiness and commitment to improving the quality of education in schools.

Despite some research on employees’ happiness and commitment [18,38,40], studies on organizational happiness and commitment in private schools are limited, and existing research has two notable gaps. First, many studies have examined organizational well-being and commitment in relation to other organizational factors [e.g., 17,21,22]. Furthermore, most studies focusing on private schools have employed either quantitative or qualitative research approaches [e.g., 12,14,15,16,17,55]. There were relatively few mixed-methods studies that delve deeply into the unique nature of private schools [e.g., 18]. Therefore, this study aims to address these gaps by examining the relationship between organizational happiness and commitment among private school teachers using a sequential explanatory mixed-methods approach, focusing on the factors influencing their happiness and commitment. Recent research indicates that overall well-being, including teachers’ happiness, influences their motivation to retain teachers in schools [e.g., 1,56,57]. The schools’ workplace climate and administrative policies play a decisive role in the emotions teachers experience. Understanding and examining various workplace-related organizational dynamics—such as a lack of recognition, adverse working conditions, and destructive attitudes and behaviors of administrators—is important and necessary for resolving organizational issues. At the same time, this highlights the need for evidence-based strategies to support and strengthen teachers’ organizational happiness and commitment. In doing so, this study contributes to literature by providing both theoretical insights and practical implications for private school administrators and policymakers. Thus, we formulated the following research questions:

### Quantitative strand

RQ1: What is the relationship between private school teachers’ organizational happiness and their commitment?
RQ2: How does private school teachers’ organizational happiness affect their school commitment?

Based on the conceptual framework and research questions above, we propose the following hypotheses for the quantitative strand:

H1: Teachers’ organizational happiness is significantly related to their affective commitment.
*H1a: Positive affect is positively related to teachers’ affective commitment.*
*H1b: Negative affect is negatively related to teachers’ affective commitment.*
*H1c: Fulfillment is positively related to teachers’ affective commitment.*
H2: Teachers’ organizational happiness is significantly related to their continuance commitment.
*H2a: Positive affect is positively related to teachers’ continuance commitment.*
*H2b: Negative affect is negatively related to teachers’ continuance commitment.*
*H2c: Fulfillment is positively related to teachers’ continuance commitment.*
H3: Teachers’ organizational happiness is significantly related to their normative commitment.
*H3a: Positive affect is positively related to teachers’ normative commitment.*
*H3b: Negative affect is negatively related to teachers’ normative commitment.*
*H3c: Fulfillment is positively related to teachers’ normative commitment*

### Qualitative strand

RQ3: What are the emotional states of private school teachers at work?
RQ4: What factors influence teachers’ happiness at school?
RQ5: What factors affect teachers’ organizational commitment in schools?

## Materials and methods

In this study, a sequential explanatory mixed-methods approach was adopted to provide a comprehensive and detailed understanding of the relationship between private school teachers’ organizational happiness and organizational commitment. While quantitative data allow testing the relationships between teachers’ organizational happiness and organizational commitment, qualitative data offer deeper insights into the contextual factors influencing teachers’ experiences regarding these issues. Additionally, this design enables the interpretation of quantitative findings in a more detailed and explanatory manner through qualitative evidence. This sequential explanatory mixed-methods study first utilized quantitative tools in a cross-sectional design, followed by qualitative tools in a case study design.

### Participants

The sample size for the quantitative strand was determined using the formula for determining the sample size for continuous data in Table 1 [58].

**Table 1:** Required Minimum Sample Size for the Quantitative Strand.

Parameters	Calculation and Values
Population	N = 592
Margin of error	d = 0,05
Estimate of standard deviation in the population	s = 0.5
The critical value corresponding to 95% confidence level	t = 1.96
Initial sample size (n_0_)	$n_{0}={\left(\frac{t\cdot s}{d}\right)}^{2}={\left(\frac{1.96\times 0.50}{0.05}\right)}^{2}=384.16$
Required sample size (n)	$n=\frac{n_{0}}{1+\frac{n_{0}}{N}}\;=\frac{384.16}{1+\frac{384.16}{592}}\approx 234$

In this regard, the minimum required sample size (with 95% confidence and a 5% error margin) was determined to be 234. Convenience sampling was used to determine the sample in this strand. First, we visited private schools and asked volunteer teachers who were available at that time to complete the paper questionnaires. Due to difficulties and constraints, teachers at schools that could not be visited were requested to complete online questionnaires. However, we contacted more teachers face-to-face or via online forms, and the final quantitative sample included 299 teachers from private schools in Alanya, Türkiye.

In addition, we performed an a priori power analysis using G*Power 3.1.9.7 for multiple linear regression analysis (F-test, fixed model, R² deviation from zero). With a medium effect size of f² = 0.15, an alpha level of 0.05, a desired statistical power of 0.95, and three predictors, the minimum sample size required was determined to be 119 teachers. The achieved sample size (n = 299) exceeded the minimum required sample size, indicating that the study possessed more than adequate statistical power to detect medium-sized effects [59].

Of the participants, 216 (72.2%) were female, and 83 (27.8%) were male. The participants’ educational levels varied, with 252 teachers (84.3%) holding undergraduate degrees and 47 (15.7%) holding graduate degrees. The participants were preschool teachers (55, 18.4%), classroom teachers (60, 20.1%), and subject teachers (184, 61.5%). Employment spans various institutions: kindergarten (55 teachers, 18.4%), primary school (60 teachers, 20.1%), secondary school (94 teachers, 31.4%), and high school (90 teachers, 30.1%). Seniority was 1–5 years (83.6%), 6–10 years (11.4%), 11–15 years (2.7%), 16–21 years (1%), and ≥21 years (1.3%).

Eight volunteer teachers from various schools and levels participated in the qualitative sample, which was selected through maximum diversity sampling method. The group comprised three men and five women, aged 25–50 years, with professional experience ranging from 3 to 20 years. Three participants held graduate degrees, and five held undergraduate degrees. The group included one preschool teacher, one classroom teacher, two Turkish language teachers, one English language teacher, one mathematics teacher, one literature teacher, and one physics teacher.

### Measures

This study used a mixed-methods sequential explanatory design, starting with a quantitative phase and followed by a qualitative phase. In the quantitative phase, two key scales were employed: a questionnaire to assess teachers’ organizational happiness and another to determine their organizational commitment levels. We employed these scales because they have demonstrated validity and reliability in previous studies [e.g., 19,23,60–62] and are suitable and are suitable for assessing organizational happiness and commitment in schools. The qualitative phase used a semi-structured interview form to explore teachers’ organizational happiness and commitment.

### Organizational happiness

The Turkish version of the Organizational Happiness Scale (OHS) [60] is based on the Well-Being at Work Scale, which was initially developed by Paschoal and Tamayo [19] to measure the organizational happiness levels of employees in Brazil. The Well-Being at Work Scale includes items on positive and negative emotions, humor, and achievement, and is divided into three factors: positive affect, negative affect, and achievement. Demo and Paschoal [23] later adapted it into English for use in the USA, maintaining the factors of positive affect, negative affect, and fulfillment. Later, in 2017, Arslan and Polat [60] adapted this scale into Turkish as the Organizational Happiness Scale to evaluate teachers’ organizational happiness.

In this study, we used the OHS adapted into Turkish by Arslan and Polat [60]. The 29-item scale with a 5-point Likert rating from “Never (1)” to “Always (5)” covers three factors: positive affect (9 items), negative affect (12 items), and fulfillment (8 items). In the current study, construct validity and factor structure were assessed via Confirmatory Factor Analysis (CFA) with 299 teacher questionnaires, yielding the following indicators: χ²/df = 2.03, RMSEA = .059, SRMR = .054, CFI = .97, and NNFI = .97. Thus, the 29-item, 3-factor structure of the OHS was confirmed as a model [63,64]. Reliability studies of the OHS revealed an item-total correlation using Cronbach’s alpha of 0.897 for positive affect, 0.904 for negative affect, 0.895 for fulfillment, and 0.832 for the overall scale.

### Organizational commitment

The “Organizational Commitment Scale (OCS)” by Meyer, Allen, and Smith [61], adapted to Turkish by Dağlı, Elçiçek, and Han [62], was employed to assess private school teachers’ organizational commitment levels. The scale comprises 18 items with a 5-point Likert scale ranging from “Never (1)” to “Always (5)” and covers three factors: affective commitment (six items), normative commitment (six items), and continuance commitment (six items).

CFA of the 299 teacher questionnaires was used to assess the construct validity and factor structure of the scale. The fit indices were χ²/df = 3.44, RMSEA = .091, SRMR = .065, CFI = .96, and NNFI = .95. Thus, the 18-item, 3-factor structure of the OCS was confirmed as a model [63,64]. The item-total test correlation coefficient (Cronbach’s alpha) was 0.821 for affective commitment, 0.822 for normative commitment, 0.741 for continuance commitment, and 0.899 for the overall scale.

### Interview form

In the qualitative strand, we conducted face-to-face interviews using a semi-structured form containing seven open-ended questions to assess teachers’ views on organizational happiness and commitment (Table 2).

**Table 2:** Interview Questions.

Research Questions	Interview Questions
RQ3	How do you feel at school?
RQ4	What brings you happiness at school? What causes your unhappiness at school? How can your happiness at school be increased?
RQ5	What factors enhance your commitment to school? What do you believe decreases your organizational commitment at school? How can private school teachers’ organizational commitment be improved?

### Procedure

This study was conducted in accordance with the Declaration of Helsinki and approved by the Ethics Committee of Alanya Alaaddin Keykubat University (88431307-050.01.04-E.2764, 20.01.2020). Prior to data collection, all participants were informed of the purpose of the study, the voluntary nature of participation, and their right to withdraw at any time. All participants were teachers aged 18 years or older; therefore, parental or guardian consent was not required for participation. Written informed consent was obtained from all participants prior to data collection in both the quantitative and qualitative phases. Confidentiality and anonymity were maintained throughout the study. To protect the participants’ privacy, no personally identifiable information was collected. In the presentation of the data, codes (e.g., T1, T2) were used for participants to ensure anonymity. All collected data were stored in secure files accessible only to the research team. The findings were reported anonymously.

The quantitative strand utilized two questionnaires: the Organizational Happiness Scale and the Organizational Commitment Scale. Volunteer teachers who were available were asked to complete the questionnaires. Of the 299 questionnaires, 88 (29.4%) were collected using a paper questionnaire. A total of 211 (70.6%) participants were asked to complete the questionnaire online owing to transportation and time constraints. Although most of the data were collected online, independent samples t-test analyses showed that online and paper-based questionnaires yielded comparable results for all variables (>.05).

Qualitative data were gathered through online (5 participants, 62.5%) and face-to-face interviews (3 participants, 37.5%) using a semi-structured interview form (Table 2). Face-to-face interviews were conducted in the participants’ school environments. Data were collected from February 17, 2020, to April 19, 2020.

In the quantitative strand, correlation and multiple linear regression (MLR) analyses were performed to examine the relationship between organizational happiness and commitment among private school teachers. We used the MLR analysis method because it allows us to examine the predictive relationships between dependent and multiple independent variables.

The simultaneous regression approach (or “Enter” as it is known in SPSS), which is a commonly used method in MLR analysis, was preferred in this study. The simultaneous regression approach is frequently used when there is no prior knowledge of the relationships between independent and dependent variables [65]. In this approach, the main objective was to examine the combined effects of all predictor variables on the dependent variable [66]. In this study, since there was no prior information obtained regarding the effects of the independent variables on the dependent variables and since the purpose was to examine the combined effects of all predictor variables on the dependent variable (as all independent variables are sub-factors of the organizational happiness scale), simultaneous regression was adopted as the basis in MLR analyses.

Before the MLR analyses, we conducted a series of preliminary analyses. First, we checked convergent and discriminant validity by calculating Cronbach’s alpha (α), average variance extracted (AVE), composite reliability (CR), and the heterotrait–monotrait ratio (HTMT).

Second, we checked the assumptions of the MLR analyses. Skewness and kurtosis values (within the range of −2 and +2 for normal distribution) were examined for normal distribution. The Durbin-Watson (D-W) test was used to assess whether there was autocorrelation in the model. A D-W value less than 1 or greater than 3 indicates a problem. To check for multicollinearity, the correlations between the independent variables were analyzed. Generally, a correlation above.80 is considered to be problematic. Additionally, Tolerance and VIF values were examined. To avoid issues, the tolerance value should not be lower than.10, and the VIF values should be less than 10 [63,67,68]. The kurtosis and skewness values of all variables were within the range of −2 to +2, indicating that the variables had normal distributions. The correlations between predictor variables were below.80, the D-W coefficient was between 1 and 3, the tolerance values were greater than.10, and the VIF values were well below 10.

In the qualitative strand, we first transcribed the audio recordings of the interviews verbatim and then analyzed the qualitative data using Braun and Clarke’s [69] six-phased thematic analysis with an inductive approach: (1) familiarization with the data, (2) coding, (3) searching for initial themes, (4) reviewing themes, (5) defining and naming the themes, and (6) writing the report. Finally, the findings were thematized based on the code relationships, and direct quotations from the participants were included to effectively present their views.

Various approaches have been used to increase the validity and reliability of qualitative data. All interviews were audio-recorded, and a thematic framework was used to ensure consistency in the data analysis. The participants reviewed the draft results to verify these findings. To ensure reliability, the researchers independently analyzed the collected data. A reliability analysis based on Miles and Huberman [70] revealed a 90.4% consistency rate between the coders. The researchers met twice to resolve any disagreements and establish common codes.

## Results

### Preliminary analysis

In the quantitative strand, we first investigated normality, reliability, convergence, and discriminant validity. Table 3 presents the normality, reliability, and validity values for all variables.

**Table 3:** Reliability and Validity Analyses.

Variables	Skewness	Kurtosis	α	CR	AVE	HTMT					
						PA	NA	F	AC	CC	NC
Independent Variables											
Positive Affect (PA)	−1.232	1.050	.897	.899	.499	–					
Negative Affect (NA)	.185	−1.252	.904	.904	.444	.286	–				
Fulfillment (F)	−1.493	1.855	.895	.896	.521	.766	.194	–			
Dependent Variables											
Affective commitment (AC)	−.780	.434	.821	.788	.411	.539	.511	.539	–		
Continuance commitment (CC)	−.449	.249	.741	.728	.332	.399	.330	.390	.678	–	
Normative commitment (NC)	−.426	−.492	.822	.835	.475	.462	.445	.449	.787	.889	–

Note: α = Cronbach’s alpha; CR = Composite reliability; AVE = Average variance extracted; HTMT = Heterotrait-monotrait ratio.

**Table 4:** Correlation Matrix between Organizational Happiness and Commitment.

Variables	M	SD	PA	NA	F	AC	CC	NC
Organizational Happiness Scale								
Positive Affect (PA)	4.07	.96	1					
Negative Affect (NA)	2.75	1.25	−.258^**^	1				
Fulfillment (F)	4.34	.87	.686^**^	−.179^**^	1			
Organizational Commitment Scale								
Affective commitment (AC)	3.90	.91	.460^**^	−.441^**^	.460^**^	1		
Continuance commitment (CC)	3.37	.83	.324^**^	−.265^**^	.314^**^	.503^**^	1	
Normative commitment (NC)	3.62	.93	.395^**^	−.377^**^	.384^**^	.630^**^	.691^**^	1

Note: n = 299; **p < .01; M: Mean scores; SD: Standard deviation.

The kurtosis and skewness values of all six variables were within the range of −2 to +2, indicating that the variables had normal distributions [68]. Reliability was ruled out through Cronbach’s alpha coefficients (ranging from.741 to.904) and CR values (ranging from.728 to.904), all exceeding the recommended threshold of.70 [63].

AVE values were investigated for convergent validity. Fulfillment (AVE = .521) exceeded the recommended threshold of.50 [71], and positive affect (AVE = .499) was marginally below this threshold. Despite the remaining variables falling below.50, we deemed the convergent validity acceptable because all CR values were well above.60 [71,72].

The three organizational happiness factors (PA, NA, and F) simultaneously served as predictors in each of the MLR models. Discriminant validity is a concern for predictor variables in MLR models. We checked the HTMT ratios, which are regarded as better than the Fornell-Larcker criterion in assessing discriminant validity violations [73]. Among the independent variables, all HTMT values were well below the threshold of.85, indicating that PA, NA, and F were empirically distinct constructs [73]. This is further supported by the VIF values in the MLR analyses, all of which were below the recommended threshold (Tables 5–7), indicating no multicollinearity concerns. The HTMT ratios between the independent and dependent variables were all below the recommended threshold of.85. This shows that the predictor and dependent variables are distinguishable.

**Table 5:** Regression Analysis of Affective Commitment.

Variable	B	SE	β	t	p	Zero-order r	Partial r	Tolerance	VIF
Constant	2.642	.264		9.992	.000				
Positive Affect	.176	.062	.185	2.840	.005	.460	.163	.510	1.961
Negative Affect	−.251	.035	−.344	−7.153	.000	−.441	−.384	.933	1.071
Fulfillment	.285	.067	.272	4.253	.000	.460	.240	.529	1.891

R = .602 R^2^ = .362.

F_(3–295)_= 55.810; p = .000.

**Table 6:** Regression Analysis of Continuance Commitment.

Variable	B	SE	β	t	p	Zero-order r	Partial r	Tolerance	VIF
Constant	2.460	.278		8.832	.000				
Positive Affect	.135	.065	.155	2.073	.039	.324	.120	.510	1.961
Negative Affect	−.129	.037	−.194	−3.501	.001	−.265	−.200	.933	1.071
Fulfillment	.166	.071	.173	2.354	.019	.314	.136	.529	1.891

R = .395 R^2^ = .156.

F_(3–295)_= 18.183 p = .000.

**Table 7:** Regression Analysis of Normative Commitment.

Variable	B	SE	β	t	p	Zero-order r	Partial r	Tolerance	VIF
Constant	2.548	.292		8.726	.000				
Positive Affect	.168	.068	.172	2.459	.015	.395	.142	.510	1.961
Negative Affect	−.220	.039	-. 294	−5.685	.000	−.377	−.314	.933	1.071
Fulfillment	.230	.074	.213	3.101	.002	.384	.178	.529	1.891

R = .511 R^2^ = .261.

F_(3–295)_= 34.748 p = .000.

The HTMT values among the dependent variables (AC–CC:.678; AC–NC:.787) were all below the.85 threshold, indicating adequate discriminant validity. However, the HTMT value between CC and NC was slightly higher than the conservative.85 threshold proposed by Henseler et al. [73]. However, this value was still below the more liberal threshold of.90 [74]. What really matters is that organizational commitment factors (AC, CC, and NC) were dependent variables and therefore regressed independently in separate MLR models. Therefore, this partial overlap is deemed not to affect the validity of the regression findings.

### Results for quantitative strand

In this section, we first examine the relationship between organizational happiness and commitment (RQ1). In the second part of this strand, we investigated how private school teachers’ organizational happiness predicts their school commitment (RQ2) and tested H1, H2, and H3 and their subsequent hypotheses through MLR analyses. Table 4 presents the descriptive statistics and correlation analysis results for the study variables.

Teachers experienced high positive affect (M = 4.07; SD = 0.96), very high fulfillment (M = 4.34; SD = 0.87), and moderate negative affect (M = 2.75; SD = 1.25). With respect to organizational commitment, teachers exhibited high affective commitment (M = 3.90; SD = 0.91), high normative commitment (M = 3.62; SD = 0.93), and moderate continuance commitment (M = 3.37; SD = 0.83) toward their schools. All the variables were significantly correlated. As anticipated, positive affect was positively correlated with affective, continuance, and normative commitments. Fulfillment was also positively correlated with all three types of commitment in this study. Conversely, negative affect was negatively correlated with affective, continuance, and normative commitments.

Multiple linear regression analysis (simultaneous approach) was used to assess whether organizational happiness factors (positive affect, negative affect, and fulfillment) significantly predicted teachers’ affective commitment (Table 5), continuance commitment (Table 6), and normative commitment (Table 7).

The model in Table 5 significantly explained 36.2% of the variance (R = .602; R² = .362; F = 55,810; p < .001). Positive affect (β = .185, p < .05), negative affect (β = −.344, p < .001), and fulfillment (β = .272, p < .001) were significant predictors of affective commitment. The relative importance of the predictors was negative affect, fulfillment, and positive affect. Fulfillment and positive affect were positively correlated with affective commitment, whereas negative affect was negatively correlated. Thereby, all H1 (H1a, H1b, and H1c) hypotheses were supported.

The model in Table 6 explained 15.6% of the variance significantly (R = .395; R² = .156; F = 18.813; p < .001). Positive affect (β = .155, p < .05), negative affect (β = −.194, p < .001), and fulfillment (β = .173, p < .05) significantly predicted continuance commitment. The predictors’ relative importance for continuance commitment was negative affect, fulfillment, and positive affect. Fulfillment and positive affect were positively associated with continuance commitment, whereas negative affect was negatively associated. Thus, all H2 (H2a, H2b, and H2c) hypotheses were supported.

The model in Table 7 explained 26.1% of the variance significantly (R = .511; R² = .261; F = 34.748; p < .001). Positive emotions (β = 0.172, p < 0.05), negative emotions (β = −0.294, p < 0.001), and fulfillment (β = 0.213, p < 0.05) were significant predictors of normative commitment. The predictors’ relative importance for normative commitment was negative affect, fulfillment, and positive affect. Fulfillment and positive affect were positively correlated with normative commitment, whereas negative affect was negatively correlated, indicating that all H3 (H3a, H3b, and H3c) hypotheses were supported.

### Results for qualitative strand

In the qualitative strand, we examined teachers’ emotions at school (RQ3) and identified the factors influencing their happiness and strategies for enhancing it (RQ4). Concurrently, we investigated the elements affecting teachers’ commitment and the measures required to increase it (RQ5).

### Teachers’ emotional states at school

The participants were first asked about their feelings at school (RQ3). The responses were categorized thematically into two groups: positive and negative affect, as shown in Table 8 below.

**Table 8:** The Emotional States of Teachers in Schools.

Themes	Codes	Participants	n
Positive Affect	Happy	T3, T4, T7, T8	4
	Energetic	T2, T8	2
	Peaceful	T2, T4	2
	Excited	T7	1
	Feels Good	T3	1
Negative Affect	Restless	T1	1
	Unhappy	T1	1
	Stressful	T5	1
	Tired	T6	1

**Table 9:** Factors that Make Teachers Happy at School.

Themes	Codes	Participants	n
Educational Activities	Teaching	T1, T3, T4, T6, T7, T8	6
	Classwork	T6, T7, T8	3
Managerial Politics	Manager recognition	T2	1
	Punctual remuneration	T3	1
Partners	Colleagues	T4, T5	2
	Parental sensitivity	T7	1
	Parental appreciation	T7	1

Most participants (n = 5) reported positive emotions upon arriving at school, including happiness, energy, peace, excitement, and general well-being. The prevailing sentiment was that the teachers appeared to be happy. For instance, T7 stated, “I feel happy when I come to school. In the morning, I do not say “I’m going to work”; I say “I’m going to school.” This is an important distinction in this study. This is because teaching is a special profession. I am excited to teach my students something new to support their development”. Similarly, S8 said, “I generally feel energetic and happy when I come to this school.”

Three participants reported negative emotions upon arriving at school, including restlessness, unhappiness, stress, and fatigue. T5 feels stressed: “During the day, when the shift starts, I am first in an intense working tempo on the axis of my responsibilities. Naturally, the mood I am in when I come to school is influenced by the stress of the workload that needs to be done that day.” T6 noted that he felt tired: “The moment I start climbing the stairs, I feel tired. I am always tired because I can not rest enough, and we have a lot of work to do at school.”

### Factors affecting teachers’ happiness at school

To identify the factors influencing teachers’ happiness at school (RQ4), the interviewees were asked three questions: What contributes to their happiness (Table 9), what causes their unhappiness (Table 10), and what can be done to increase their happiness (Table 11)? The interviewees detailed the elements that made them happy at school, as shown in Table 9.

**Table 10:** Factors that Make Teachers Unhappy at School.

Themes	Codes	Participants	n
Managerial Approach	Managerial attitude	T1, T8	2
	Excessive expectations	T6	1
	Gossiping managers	T1	1
	Unfair managerial evaluation	T2	1
	Manager pressure	T5	1
	Unappreciative managers	T6	1
Colleagues	Selfish colleagues	T8	1
	Unemphatic colleagues	T8	1
	Being ignored for their opinions	T7	1
	Collegial rivalry	T8	1
	Inadequate colleagues	T3	1
Working Conditions	Unnecessary school rules	T8	1
	Excessive workload	T6	1
Parental Attitudes	Excessive parental demands	T6	1
	Parental intervention	T4	1

**Table 11:** Things that can be Done to Increase Teacher Happiness in Schools.

Themes	Codes	Participants	n
Motivation	Giving value	T2, T3, T5, T8	4
	Financial improvements	T5, T6	2
	Social activities	T3	1
	Rewards	T2	1
	Appreciation	T2	1
	Respect	T7	1
	Parental support	T4	1
Workload	Reduced work hours	T1, T3	2
	Fair share of duties	T2	1
	Joker teacher	T2	1
Professional Development	Professional development opportunities	T2	1
	Professional support	T4	1
Managerial Approach	Not to convey teacher problems to others	T1	1
	Clear job description	T6	1
	Giving importance to teachers’ problems	T1	1

We identified three themes contributing to teachers’ happiness: educational activities (n = 6), managerial politics (n = 2), and partners (n = 3). Most participants indicated that teaching and classwork made them happy. For example, T6 remarked, “The only thing that makes me happy at school is going to class, teaching, and doing things related to teaching.”

Other factors that make teachers happy include punctual remuneration and management’s recognition. We refer to this as managerial politics. Interviewee T3 highlights the impact of economic factors: “In addition, I am happy that salaries are paid on time, that insurance is paid properly, and that we are treated fairly.”

During the interviews, the teachers highlighted that parental sensitivity and appreciation, along with their colleagues’ support, contributed to their overall happiness. T7 expressed happiness due to parents’ sensitivity: “We are educators, and we are happy to see parents who care about their children’s education. For example, it makes me happy that parents immediately buy the books we recommend for their children, that they want to improve their children, and that we are in good solidarity on this path.”

In addition, the participants were asked to identify the factors causing their unhappiness at school. The results are presented in Table 10.

Opinions were collected under four themes: managerial approach (n = 5), colleagues (n = 3), working conditions (n = 2), and parental attitudes (n = 2). Five interviewees reported that a negative managerial approach caused unhappiness, citing issues such as the administrator’s attitude, administrative expectations, gossip, unfair evaluations, oppression, and a lack of appreciation. Similarly, five interviewees were unhappy for colleague-related reasons. Additionally, two teachers attributed their unhappiness to negative parental attitudes and poor working conditions. The most frequently cited sources of unhappiness were managerial approaches and colleague-based factors. For example, T2 noted that she and her colleagues were unhappy due to unsupportive administrators, stating, “Given that I work in a private school, it is sad that the administrators respond to the parents’ words instead of supporting the teachers, and the failure of the students is blamed on us.” T7 mentioned that her unhappiness stemmed from a lack of appreciation from colleagues: “The new ideas that I have proposed in some meetings have not been given much importance by other teachers.” T1 also reported unhappiness due to the lack of a positive school atmosphere: “There is too much gossip in our school.”

Third, we asked the participants, “What can be done to increase your happiness in school?” Their suggestions are presented in Table 11.

The factors that can increase teacher happiness in schools were identified in four themes: motivation (n = 7), workload (n = 3), professional development (n = 2), and managerial approach (n = 2).

Seven participants emphasized the need for motivation to increase their happiness. They suggested that managers or founders should make teachers feel valued, improve their financial conditions, organize social activities, establish an effective reward system, and show appreciation and respect for teachers. Additionally, parental support was considered crucial. For example, T5 highlighted the importance of feeling valued: “There are two basic steps to increase teachers’ happiness and work performance at school: knowing and feeling that they are valued.” T2 and T8 echoed this sentiment, noting that attention to teachers’ lives and valuing their opinions increased happiness: “Teachers’ opinions should also be valued.” (T8), “Administrators should keep track of teachers’ special days. Seeing value in the workplace is the most important factor that increases happiness” (T2).

Two participants highlighted workload as a key factor in enhancing teachers’ happiness. They proposed reducing teachers’ workloads and working hours, ensuring fair task distribution among colleagues, and implementing a joker-teacher model. The most emphasized suggestion was to reduce working hours. T3 mentioned, “Maybe reducing the class hours or decreasing the workload,” and T1 similarly said, “Working hours can be reduced a little.” Additionally, T3 recommended employing joker teachers: “A substitute teacher or an assistant teacher can be recruited for each subject. They can put teachers at ease.” He stressed that teacher happiness can be improved by increasing the number of teachers to an appropriate level.

The interviews revealed that professional development opportunities positively affect the organizational happiness. T2 noted that acquiring essential knowledge, skills, and behaviors enhances effectiveness and productivity, leading to happiness, as exemplified by the statement, “Providing opportunities for teacher development by in-service training.”

Moreover, the interviewees emphasized the impact of a positive managerial approach on teachers’ happiness, including not burdening others with teacher problems, providing clear job descriptions, and addressing teacher issues. For instance, T6 mentioned that clear expectations enable teachers to fulfill their responsibilities better and be happier: “Job descriptions need limitations. As teachers, you do a lot of work, not just teaching. You perform guidance services, vice principals’ tasks, and sometimes even the principal’s duties. This is too exhausting. Teachers must constantly perform their own tasks as well as other tasks. Job descriptions for teachers should be clarified. I think this can make teachers happy.” Thus, teachers with clear responsibilities may be more successful and beneficial because of their effective teaching. Another teacher, T1, noted that administrators who transferred their problems to colleagues or parents harmed their work environment. She stressed that only a trusted administrator could increase teacher happiness: “Teacher-related issues should only be discussed with the concerned individual and not transferred to second or third persons.”

### Factors affecting teachers’ commitment to their schools

To identify the factors influencing teachers’ organizational commitment to their schools (RQ5), three questions were posed: (1) What factors enhance your commitment to the school? (2) What do you believe decreases your organizational commitment to your school? (3) How can private school teachers’ organizational commitment be improved?

We investigated the factors that enhance teachers’ organizational commitment to their schools (Table 12). The key themes identified were managerial approaches, financial benefits and social relationships.

**Table 12:** Factors that Increase Teachers’ Organizational Commitment.

Themes	Codes	Participants	n
Managerial Approach	Freedom of thought	T2	1
	Delegation of authority	T2	1
	Fair Administration	T8	1
	Shared mission and vision	T5	1
	Easy permission	T4	1
	Reduction of duties	T8	1
Financial Issues	Enhanced economic circumstances	T1, T4, T6	3
	Timely remuneration	T7	1
	Need for employment	T1	1
Social Relationship	Solidarity	T3, T6	2
	Confidence	T3	1
	Collaborative relationship	T3	1
	Gratefulness	T4	1

The interviews (n = 4) indicated that the administrators’ attitudes and behaviors enhanced their organizational commitment. Predominant factors increasing their commitment were freedom of thought, delegation of authority, fair administration, shared school missions and vision, easy permission procedures, and reduced work duties. For instance, T2 mentioned that administrators who delegate authority and responsibility to teachers can strengthen organizational commitment: “I think that the administrators sharing their duties, authorities, and responsibilities with us increases our commitment.”

With respect to financial issues, the participants (n = 4) indicated that timely salary payments, the need for employment, and improvements in economic conditions, such as higher earnings, would increase their organizational commitment. T4 attributed his commitment to high financial gain: “When we talk about this issue with my colleagues, I see that they mostly think that financial issues increase organizational commitment. A teacher who is financially satisfied becomes more committed to her/his school.”

Furthermore, three interviewees suggested that enhancing solidarity, trust, cooperation, and gratitude could improve social relations within the school and subsequently increase organizational commitment. T4 highlighted that behaviors fostering gratitude can increase a teacher’s affective commitment to the school, citing supportive actions such as helping and providing opportunities: “Behaviors that make a person grateful can also increase a teacher’s commitment to the school. I think that helping more, providing more opportunities, and giving permission in our private affairs or in special situations will increase our organizational commitment.”

With respect to reducing their organizational commitment to schools, the interviewees stated three fundamental factors: negative managerial approaches, negative school policies, and destructive social relations (Table 13).

**Table 13:** Factors that Decrease Teachers’ Organizational Commitment.

Themes	Codes	Participants	n
Negative Managerial Approach	Adverse manager attitudes	T1, T4, T6	3
	Underrating teachers	T4, T7	2
	Not being appreciated	T2, T5	2
	Lack of justice Inequitable treatment	T7	1
Negative School Policy	Low salary	T2, T3, T5	3
	Excessive workload	T3, T5	2
	Undue parental interference	T2	1
Destructive Social Relationships	Gossip	T1	1
	Lack of professional respect	T7	1

The interviewees (n = 6) indicated that negative managerial attitudes were the primary reason for diminished organizational commitment among teachers. They felt that administrators were unfair, undervalued teachers, ignored their opinions, did not appreciate their work, and held negative attitudes toward them. T4 expressed discomfort with managerial attitudes, stating, “Not supporting the teacher in even any small thing can reduce organizational commitment.” T2 emphasized the importance of appreciation: “Appreciation increases the commitment, but vice versa, not being appreciated decreases it.” T6 noted that the absence of equality and justice and mistrust in administrators led to reduced commitment and unhappiness: “There is no justice among teachers. Who worked more or fewer hours? Who has the highest and lowest salary? They have no criteria, and people feel worthless. This reduces commitment to the organization. Anyone can give up and leave whenever they want, and this is happening right in front of us in the current study. We often observe examples of this phenomenon. This is why the staff constantly changes. Everyone is changing. This demotivates people.”

Additionally, three interviewees highlighted that negative school policies reduce teachers’ organizational commitment. The major issues include low salaries, long working hours and undue parental interference. T2 explained, “If you are a private sector employee, not receiving financial compensation reduces commitment.” Similarly, another interviewee, T5, pointed to low income as a major factor for decreasing commitment: “Low salary is the main factor for me.” Long working hours also caused reduced commitment, as T3 noted, “For some teachers, this work pace may be heavy. The workload is too much.”

Two participants cited destructive social relationships as factors associated with low commitment levels. Gossip and lack of respect were prevalent issues. For example, T7 explained, “The new ideas put forward in some meetings were not given much importance and were not respected by other teachers.” The absence of positive social relationships and feelings of being undervalued by colleagues led to decreased commitment to the institution.

Moreover, we asked the participants how their organizational commitment to school could be enhanced. Table 14 outlines the teachers’ suggestions for increasing this commitment.

**Table 14:** Several Measures can be taken to Increase Organizational Commitment.

Themes	Codes	Participants	n
Managerial Approach	Rewards	T6, T8	2
	Not to gossip	T1	1
	Boosting teacher happiness	T6	1
	Appreciation for teachers	T5	1
	Fostering respect for teachers	T1	1
	Limiting nonpedagogical responsibilities	T4	1
	Positive administrator practices	T1	1
	Valuing teacher ideas	T8	1
	Incorporating teachers in decision-making process	T2	1
Working Conditions	Reduced workload	T3, T4, T5	3
	Enhanced weekly teaching schedules	T2, T5	2
	Optimizing leave hours of teachers	T2, T4	2
	Equitable distribution of responsibilities	T3	1
	Enhancing educator benefits	T4	1
Economic Conditions	Monetary rewards	T4, T6, T8	3
	Economic benefit	T4, T7	2
Social Relations and Activities	Motivation-enhancing activities	T5, T8	2
	Improving social infrastructure	T5, T6	2
	Consideration of ideas	T1	1
	Organizing social events for teachers	T5	1

The key factors that increase organizational commitment include the managerial approach (n = 6), working conditions (n = 4), economic conditions (n = 4), and social relations and activities (n = 3), with managerial attitudes being the most emphasized.

Teachers (n = 6) noted that managerial attitudes, such as rewarding teachers, avoiding gossip, valuing teacher happiness, appreciation, respect, limiting non-pedagogical tasks, positive administrative practices, considering teacher opinions, and involving teachers in decision-making, increase commitment among teachers. Teachers emphasized that school managers must exhibit attitudes and behaviors that foster teachers’ organizational commitment. As T1 stated, “They could be more sincere. They should not talk behind their teachers’ backs. These apply to me and my school. However, this may not have been the case in other schools. At the school where I currently work, my principal can gossip about another teacher with a teacher or use that teacher’s discourse. He can show a hostile attitude toward his teacher.”

In addition, four teachers proposed that enhanced working conditions, such as reduced workload, optimized weekly teaching schedules, fair distribution of responsibilities, and improved benefits, would increase their commitment. T3 stated that reducing the course load by hiring additional teachers would increase commitment: “It may be having additional teachers in a way that will reduce the course load.” Similarly, T4 highlighted the importance of half-days off, mentioning their successful implementation in their school and the teachers’ dissatisfaction when it was suggested to remove them by stating, “Setting the days off, that is, half a day off, can be allowed. Our school has implemented this program in the past. Everyone is satisfied. It has even been suggested that days off should be eliminated. The teachers were dissatisfied with the training provided. We can handle our official affairs on those days, or those who want to rest can rest.” T2 also emphasized the importance of collaboratively adjusting weekly lesson plans with teachers and offering flexibility in the number of lessons or half-days off to improve organizational commitment: “To increase organizational commitment, the weekly lesson plan can be adjusted together with the teachers. It would also be good to give teachers half a day off or to offer flexibility in the number of lessons.”

Four teachers stressed that favorable economic conditions, such as regular salary payments, fostered organizational commitment. T7 emphasized, “Regular payment of salaries is a commitment-boosting thing.”

With respect to social relations, teachers (n = 4) recommended motivation-enhancing activities, improved social infrastructure, consideration of ideas, and more social activities to enhance organizational commitment. In addition, T5 said, “Teacher social life areas should be established, and motivational activities organized to increase commitment.”

## Discussion

Using a sequential explanatory mixed-methods approach, this study revealed the relationship between teachers’ organizational happiness and organizational commitment, with a particular focus on the factors that enhance and reduce happiness and commitment.

The quantitative results provide a general understanding of the relationship between organizational happiness and commitment among private school teachers. Teachers reported high positive affect and fulfillment with less negative affect, which was less prevalent than expected. Teachers showed high affective and normative commitment but were uncertain about their continuance commitment. Despite their strong affective and normative commitment, their intentions to stay in their schools were unclear, which, as suggested by Meyer and Allen [75], could negatively impact employment policies in private schools because of job insecurity.

Regarding the relationship between organizational happiness and commitment, positive affect was significantly and positively correlated with affective, continuance, and normative commitments. Similarly, fulfillment showed a significant positive correlation with these types of commitments. Consequently, school administrators should attempt to create a positive work climate to enhance teachers’ commitment by promoting positive affect. This finding is corroborated by studies that indicate a strong positive relationship between organizational happiness and commitment [76]. Previous studies [77,78] have also reported a significant relationship between well-being and organizational commitment. For example, Yin, Tam, and Lau [36] found that teachers’ psychological well-being enhanced their commitment, indicating that happy teachers are more committed. In addition, this current study also revealed that negative affect had a significant negative correlation with affective, continuance, and normative commitments, suggesting that negative affect decreases teachers’ commitment. Overall, our findings suggest that school leaders need to consider teachers’ happiness measures to further increase teachers’ commitment.

Multiple regression analyses revealed that all three organizational happiness factors (positive affect, negative affect, and fulfillment) significantly predicted affective, continuance, and normative commitments. This finding is consistent with previous studies that employee well-being is a significant predictor of organizational commitment [79–81]. As Li et al. [82] noted, teachers with a high level of well-being are likely to have a stronger emotional attachment to their schools, which in turn may result in a higher affective commitment to the school. Similarly, teachers’ well-being exhibits a positive influence on their affective commitment and normative commitment [83]. In our study, organizational commitment, encompassing affective, normative, and continuance commitments, was positively correlated with positive affect and fulfillment but negatively correlated with negative affect. The most influential predictors of affective, continuance, and normative commitment were negative affect, fulfillment, and positive affect, respectively. To enhance private school teachers’ organizational commitment, school leaders should first minimize negative emotions in schools, create environments for potential realization, and support the climate to enhance their happiness at work.

Importantly, the finding that positive affect is significantly associated with affective commitment is consistent with Fredrickson’s [41] Broaden-and-Build Theory of Positive Emotions. According to this theory, positive emotions such as joy, interest, and satisfaction enable people to expand their immediate repertoire of thoughts and actions, thereby fostering lasting personal resources, including social bonds and psychological resilience. From the perspective of private school teachers, teachers who experience positive emotions may develop a stronger emotional bond with their schools because these positive emotions foster more meaningful relationships with colleagues and a deeper sense of belonging. Indeed, this mechanism is supported by our qualitative findings, which indicate that happiness derived from educational activities and peer support strengthens teachers’ sense of commitment to their schools. This result is also consistent with the study of Kun and Gadanecz [8] which reveal that the implementation of interventions to improve optimism and positive emotions may increase happiness.

However, according to Fredrickson’s [41] Broaden-and-Build Theory of Positive Emotions, negative emotions such as anxiety, stress, and disappointment can, on the contrary, narrow teachers’ cognitive and behavioral repertoires, thereby limiting their ability to engage constructively with their schools. This explains the significant negative influence of negative affect on affective, normative, and continuance commitment, which is one of the key findings of our study. Furthermore, the qualitative findings from our study also support this mechanism: teachers exposed to negative administrative attitudes—such as unfair evaluations, gossip, and a lack of recognition—reported a decrease in their emotional bonds and a weakening of their commitment.

The qualitative results supported the quantitative findings, explaining the high organizational happiness levels across all three factors. Teachers reported positive emotions at school, such as happiness, energy, excitement, and peace. Teachers with negative emotions feel restless, unhappy, stressed, and fatigued at school. Happiness increases commitment to school [40] and lowers turnover rates [37], whereas stress reduces it [84]. Moreover, negative emotions can adversely affect teachers’ work performance, relationships, tolerance, understanding, error rates, health and retirement [85].

The interviews revealed that teachers experience the greatest happiness in educational activities, such as teaching, lecturing, classroom work, and building student relationships. Appreciation from administrators and timely salary payments increase happiness. Teachers with prompt wage disbursements, correctly paid insurance premiums, and a fair and friendly workplace reported higher happiness levels, highlighting the importance of economic factors. As Gkliati et al. [86] contend, wages are a significant predictor of employee well-being, indicating that increased wages enhance employee morale and overall well-being. Furthermore, a fair and friendly atmosphere in the workplace contributes to the development of positive emotions among employees [87], which are essential determinants of happiness. Additionally, parental appreciation positively impacted teachers’ happiness. Grateful parents enhance teachers’ emotional well-being. Ensuring teachers’ job satisfaction is essential for improving educational quality and workplace happiness [88]. Moreover, a positive work environment, employee satisfaction, and positive relationships are essential for happiness in organizations, which, in turn, improves employee well-being and performance [89].

Nevertheless, destructive administrative attitudes, poor working conditions, and undesirable behaviors of colleagues and parents contribute to teachers’ dissatisfaction. Predominantly, negative attitudes from administrators, such as gossip, unfair evaluations, oppression, and lack of appreciation, are major sources of unhappiness. Parental involvement in the educational process exacerbates this issue. Teachers in the private education sector face excessive parental demands, and administrators often act on these demands, holding teachers accountable for students’ failures. The attitudes of parents and administrators create a negative working environment for teachers.

Additionally, teachers’ negative relationships with their colleagues contribute to their unhappiness. Teachers in unsupportive environments who lack empathy and mutual understanding feel undervalued and unhappy. To increase teacher happiness, it is essential to increase motivation, offer rewards, ensure fair treatment, reduce workload, provide professional development opportunities, and foster positive attitudes among parents and administrators. Bulut [90] reported that selecting meritorious administrators and improving salaries increased teachers’ happiness and satisfaction levels. Argon [91] noted that when administrators neglect teachers’ feelings, it leads to poor performance, diminished effort, reduced motivation, the intention to quit, and unhappiness.

Committed and satisfied teachers are increasingly recognized and valued as pivotal partners in enhancing school effectiveness [55]. Qualitative interviews further revealed that meritocratic administrators, higher incomes, good social relations, and favorable working conditions enhance teachers’ organizational commitment. Administrators’ positive attitudes and behaviors are the most crucial factors in increasing commitment. Organizational commitment is related to occupational commitment. Enhancing teachers’ occupational commitment helps improve their commitment to the organization. Furthermore, teachers’ commitment to their work significantly affects the quality of teaching and educational effectiveness in schools [92]. At this point, school leaders can create a supportive environment that encourages collaboration, sharing, and discussion of professional learning experiences, which, in turn, benefits teacher commitment. Teachers’ organizational commitment can be increased if administrators understand their feelings, thoughts, and expectations; provide a platform for freely expressing ideas; increase their responsibilities; foster a sense of belonging; manage workloads fairly; and involve teachers in decision-making. Teachers in private schools indicated that higher salaries upon signing contracts enhanced their commitment to the school. Social relationships, feelings of gratitude, trust in colleagues, cooperation, and solidarity contribute to this commitment. Balay [50] reported that work experience is strongly related to affective commitment. Positive work experiences increase employees’ organizational commitment and reduce absenteeism and membership termination [93].

Negative administrative attitudes, school policies, and social relations diminish commitment, with negative managerial attitudes being the most frequently mentioned issue. Factors such as perceived unfair treatment, undervaluation of teachers’ contributions, disregard for their input, and lack of appreciation for their efforts contributed to this decline. Teachers expect support, appreciation, motivation, and fair treatment from their administrators. Otherwise, perceived injustice and devaluation may lead to dissatisfaction and reluctance to work for employees. Turan [94] reported that negative managerial activities and attitudes adversely affect employee happiness. Harter and Adkins [95] reported that half of employees leave their jobs because of their managers’ negative attitudes and behaviors. Low commitment is also linked to parental interference, low pay, unfair pricing policies and long working hours. Compared to public school teachers, private school teachers face greater workloads and stress due to longer working hours, further diminishing their organizational commitment. Additionally, destructive social relationships among teachers, such as colleagues not listening to each other and workplace gossip, can weaken commitment.

Teachers commonly desire that their colleagues be sensitive to their feelings, opinions, and suggestions. Teachers who are happy at school and develop positive attitudes also derive satisfaction from their societal relationships [96]. Additionally, employees who are happy at work are more productive and strive to provide better services, thus revealing their potential [86,97]. Conversely, negative emotions at work reduce employees’ performance and productivity [91]. As Fredrickson [41] noted in her Broaden-and-Build Theory of Positive Emotions regarding the undoing hypothesis, positive emotions in schools can mitigate or eliminate the lasting effects of teachers’ negative emotions. Ultimately, it is crucial to develop policies that will make teachers happy and for school leaders to implement practices that contribute to teachers’ well-being.

## Conclusion

By integrating quantitative and qualitative findings, this study enhances organizational happiness and commitment in private schools by providing evidence to increase these attributes among the teachers. The quantitative findings indicate that organizational commitment among private school teachers can be increased by enhancing their happiness and creating environments that enable potential realization while reducing negative feelings in the workplace. The qualitative findings offered detailed descriptions of teachers’ views on organizational happiness and commitment, highlighting the factors that enhance or reduce these aspects. These findings confirm the quantitative results, showing that the factors influencing organizational happiness are linked to organizational commitment.

Quantitative research has shown that teachers have high scores for positive affect and fulfillment with respect to organizational happiness. This finding is supported by qualitative findings that teachers experience more positive affect in their schools. Conversely, teachers had relatively low scores for negative affect, corroborated by qualitative findings indicating lower negative affect than positive affect. The quantitative results suggest that positive affect is associated with increased commitment and loyalty. Qualitative analysis revealed that positive emotions such as energy, peace, excitement, well-being, and enjoyment of work enhanced organizational commitment. These emotions are linked to valuing employees, financial improvements, motivating activities, rewards, appreciation, reduced working hours, and fairness. Negative affect is likely to reduce commitment, with negative emotions stemming from managerial attitudes, gossip, unfair evaluations, fear of administrators, excessive workloads, and unnecessary rules. Similar factors influence both happiness and commitment, highlighting the importance of identifying the factors related to happiness that affect commitment. Consequently, emotions such as enthusiasm, peace, excitement, well-being, work enjoyment, higher income, and appreciation increase organizational commitment by increasing teachers’ happiness.

## Limitations and future research

This study had several limitations that should be considered in future studies. First, longitudinal studies can provide deeper insights into changes in organizational happiness and commitment over time. However, the use of a mixed-methods research design in this study may mitigate this limitation. Second, survey and interview data can be enhanced through observations. Third, this study focused on the relationship between teachers’ organizational happiness and commitment in private schools. Future research should examine the effects of perceived organizational fairness, income, and leadership practices on teachers’ happiness and commitment to their work. Similar studies in various educational settings could help validate these results.

This study has both theoretical and practical implications for improving teachers’ happiness and commitment. This study highlights the Broaden-and-Build Theory of Positive Emotions, pointing to the theory’s applicability not only in an individual context but also in an organizational context. While Fredrickson’s [41] theory focuses on how positive emotions foster individual psychological resources, our findings indicate that this mechanism also operates at the organizational level in private schools and helps increase commitment.

At the same time, our findings have practical implications for private school leaders. As highlighted in the Global Report on Teachers [5], it is well established that improving teachers’ working conditions has a significant impact on increasing and sustaining teacher recruitment. Our qualitative findings indicate that teachers perceive feeling valued, receiving fair compensation, and working under supportive leadership as the primary factors influencing both their happiness and their commitment. This is precisely why, school leaders should make employees feel valued, ensure a comfortable and happy work environment, improve working conditions, consider teachers’ suggestions and complaints, address their problems individually, and reward their achievements. Thus, teachers’ positive feelings toward their schools and the realization of their potential can increase their organizational commitment.

With respect to their happiness at school, teachers are expected to feel valued, experience positive administrative behavior, and receive supportive parental attitude. Valuing employees is deemed particularly crucial. Teachers indicated that they would be happier if they felt valued by their schools. Thus, appreciating and respecting teachers, reducing their workload, and providing a fair working environment are essential for enhancing happiness in school. In addition, future research should investigate why private school teachers are undecided about continuing to work in their schools despite their high emotional and normative commitments.

## References

[1] Stark K, Daulat N. Teacher well-being and teacher retention: establishing a link. Soc Psychol Educ. 2025;28(1). doi: 10.1007/s11218-025-10109-6

[2] Capone V, Petrillo G. Mental health in teachers: Relationships with job satisfaction, efficacy beliefs, burnout, and depression. Curr Psychol. 2020;39(6):1757–66. doi: 10.1007/s12144-018-9878-7

[3] Tsang KK. Teachers as disempowered and demoralised moral agents: School board management and teachers in Hong Kong. Br J Educ Stud. 2019;67(2):251–67. doi: 10.1080/00071005.2018.1497770

[4] Sampayo C, Leichtman K. A concern for the teaching profession: impacts of demoralization. FAU Undergrad Res J. 2021;10:21–4.

[5] UNESCO. International task force on teachers for education 2030. Global report on teachers: Addressing teacher shortages and transforming the profession. Paris: United Nations Educational, Scientific and Cultural Organization; 2024.

[6] Dreer B. Teachers’ well-being and job satisfaction: The important role of positive emotions in the workplace. Educ Stud. 2021;50(1):61–77. doi: 10.1080/03055698.2021.1940872

[7] Kolodinsky RW, Ritchie WJ, Kuna WA. Meaningful engagement: Impacts of a “calling” work orientation and perceived leadership support. J Manag Organ. 2017;24(3):406–23. doi: 10.1017/jmo.2017.19

[8] Kun A, Gadanecz P. Workplace happiness, well-being and their relationship with psychological capital: A study of Hungarian teachers. Current Psychology. 2022;41(1):185–99. doi: 10.1007/s12144-019-00550-0

[9] Diener E. Subjective well-being. The science of happiness and a proposal for a national index. Am Psychol. 2000;55(1):34–43. doi: 10.1037/0003-066x.55.1.34 11392863

[10] Salas-Vallina A, Alegre J. Happiness at work: Developing a shorter measure. J Manag Organ. 2021;27(3):460–80. doi: 10.1017/jmo.2018.24

[11] Virtanen V, Hailikari T, Murtonen M, Parpala A, Postareff L. What challenges higher education teachers’ teaching-related wellbeing?. Teach Dev. 2025;:1–19. doi: 10.1080/13664530.2025.2537056

[12] Çimen B, Karadağ E. A qualitative study on working conditions and future anxieties of teachers at private schools. İnönü Univ J Fac Educ. 2020;21(2):518–41. doi: 10.17679/inuefd.476428

[13] Brady J, Wilson E. Comparing sources of stress for state and private school teachers in England. Improv Sch. 2022;25(2):205–20. doi: 10.1177/13654802211024758

[14] Dinç M. The effect of work overload and family demands on work-family conflict: A study on private school teachers. J Organ Behav Rev. 2021;3(1):45–72.

[15] Türkoğlu ME, Aypay A. Private school teachers’ thoughts about teacher accountability. J Educ Policy Anal. 2015;4(1):7–32.

[16] Karaköse T, Kocabaş İ. The effect of teachers’ expectations on job satisfaction and motivation in private and public schools. J Theory Pract Educ. 2006;2(1):3–14.

[17] ÜLKER S, GİZİR S. Teachers’ Emotional Labor Behaviors: The Role of Positive Psychological Capital and Organizational Commitment. Mersin Üniversitesi Eğitim Fakültesi Dergisi. 2023;19(2):203–26. doi: 10.17860/mersinefd.1274890

[18] Karadaş H, Akın MA. Examining teachers’ organizational happiness and organizational commitment. E-Int J Educ Res. 2023. doi: 10.19160/e-ijer.1247022

[19] Paschoal T, Tamayo A. Construção e validação da Escala de bem-estar no trabalho. Aval Psicol. 2008;7(1):11–22.

[20] Warr P. Searching for happiness at work. Psychol. 2007;20(12):726–9.

[21] Çetin S. The relationship between secondary school teachers’ organizational justice perception levels and organizational happiness levels [master’s thesis]. Kocaeli University; 2019.

[22] Arslan Y. The relationship between teachers’ perceptions of diversity management approaches and their perceptions of organizational happiness [doctoral dissertation]. Kocaeli University; 2018.

[23] Demo G, Paschoal T. Well-Being at Work Scale: Exploratory and Confirmatory Validation in the USA. Paidéia (Ribeirão Preto). 2016;26(63):35–43. doi: 10.1590/1982-43272663201605

[24] Kuvvet AB. The relationship between principals’ instructional leadership and classroom teachers’ organizational happiness [master’s thesis]. Gazi University; 2019.

[25] Zhi Y, Derakhshan A. An Investigation into Chinese English Teachers’ Organizational Commitment: Do Emotion Regulation and Workplace Buoyancy Matter?. Asia-Pacific Edu Res. 2024;34(2):689–98. doi: 10.1007/s40299-024-00888-5

[26] Klein HJ, De Aguiar Rodrigues AC, Zhan Y. Community and organizational commitment: Understanding the effects of organizational investments on worker behavior. J Bus Res. 2024;186:115023. doi: 10.1016/j.jbusres.2024.115023

[27] Gündoğan T. Job satisfaction and organisational commitment: An application in a human resources department. Ankara University. 2010.

[28] Allen N, Meyer J. Affective, Continuance, and Normative Commitment to the Organization: An Examination of Construct Validity. J Vocat Behav. 1996;49(3):252–76. doi: 10.1006/jvbe.1996.0043 8980084

[29] Gürkan Ç. Organizational commitment: The effect of organizational climate on organizational commitment and the relationship between organizational climate and organizational commitment at Trakya University. Trakya University. 2006.

[30] Bakan İ. Örgütsel stratejinin temeli örgütsel bağlılık: Kavram, kuram, sebep ve sonuçlar. Ankara: Gazi Kitapevi. 2018.

[31] Meyer JP, Parfyonova NM. Normative commitment in the workplace: A theoretical analysis and re-conceptualization. Hum Resour Manag Rev. 2010;20(4):283–94. doi: 10.1016/j.hrmr.2009.09.001

[32] Benawa A, Gea AA, Willyarto MN. Improving teacher’s commitment by improving external factors. Adv Sci Lett. 2017;23(2):925–8. doi: 10.1166/asl.2017.7440

[33] Dertlioğlu S. Examining the relationship between organizational commitment and job satisfaction of education sector employees. İstanbul Gelişim University. 2016.

[34] Carver C. Pleasure as a sign you can attend to something else: Placing positive feelings within a general model of affect. Cogn Emot. 2003;17(2):241–61. doi: 10.1080/02699930302294 29715724

[35] Fisher CD. Happiness at work. Int J Manag Rev. 2010;12(4):384–412. doi: 10.1111/j.1468-2370.2009.00270.x

[36] Yin H, Tam WWY, Lau E. Happy teachers are efficacious and committed, but not vice versa: Unraveling the longitudinal relationships between Hong Kong kindergarten teachers’ psychological well-being, self-efficacy, and commitment. Teach Teach Educ. 2023;123:103997. doi: 10.1016/j.tate.2022.103997

[37] Yang C-C, Fan C-W, Chen K-M, Hsu S-C, Chien C-L. As a Happy Kindergarten Teacher: The Mediating Effect of Happiness Between Role Stress and Turnover Intention. Asia-Pacific Edu Res. 2018;27(6):431–40. doi: 10.1007/s40299-018-0403-4

[38] Bahat İ, Işık M. The relationship between teachers’ school happiness, organizational commitment, and self-efficacy. J Hum Soc Sci. 2023;6:279–308. doi: 10.1166/asl.2017.7440

[39] Huang W, Zhang S, Li H. Effects of person-job fit on occupational commitment among kindergarten teachers: occupational well-being as mediator and perceived organizational support as moderator. BMC Psychol. 2023;11(1):402. doi: 10.1186/s40359-023-01441-7 37986096 PMC10658734

[40] Arman M, Tan S. A study on the relationship between organizational commitment and happiness levels. Pamukkale University. 2018.

[41] Fredrickson BL. The role of positive emotions in positive psychology. The broaden-and-build theory of positive emotions. Am Psychol. 2001;56(3):218–26. doi: 10.1037//0003-066x.56.3.218 11315248 PMC3122271

[42] Fredrickson BL. The broaden-and-build theory of positive emotions. Philos Trans R Soc Lond B Biol Sci. 2004;359(1449):1367–78. doi: 10.1098/rstb.2004.1512 15347528 PMC1693418

[43] MEB. National education statistics: Formal education 2024/‘25. A Publication of Official Statistics Programme; 2025.

[44] Cerev G, Çoşkun S. A qualitative research on worklife based problems of private school teachers: the case of Elazig province. J Harput Stud. 2020;7(13):125–42.

[45] Erbay E, Özcan A. Özel okullarda görev yapan öğretmenlerin kurum politika ve uygulamalarına yönelik karşılaştığı sorunlar ve çözüm önerileri. Prem e-J Soc Sci. 2024;8(49):1619–41. doi: 10.5281/zenodo.14580332

[46] Ergen H, Çokkeser F. Private schools in Turkey: Problems encountered by teachers. Int Innov Educ Res. 2022;2(1):111–36.

[47] Aslam A, Kamran M, Siddiqui S. Job crafting and teachers’ well-being: a case of private school teachers. BMC Public Health. 2025;26(1):239. doi: 10.1186/s12889-025-25945-6 41387813 PMC12821878

[48] Oad L, Niazi S. Effects of the organizational factors on teachers’ retention: perceptions of private secondary school teachers of Lyari Town. Pak J Educ Res. 2021;4(1):214–31. doi: 10.52337/pjer.v4i1.150

[49] Kesbiç K, Korlu Ö, Gencer EG, Terzi GN, Arık BM. Eğitim izleme raporu 2024. Eğitim Reformu Girişimi; 2024. Available from: https://www.egitimreformugirisimi.org/egitim-izleme-raporu-2024

[50] Balay R. Yönetici ve Öğretmenlerde Örgütsel Bağlılık [Organizational Commitment in Managers and Teachers]. Ankara: Pegem Akademi; 2014.

[51] Sabuncuoğlu Z, Tüz M. Örgütsel Psikoloji [Organizational Psychology]. Bursa: Ezgi Kitabevi; 2001.

[52] Ergün E, Nartgün ŞS. Adaptation of teacher subjective well-being scale into Turkish: validity and reliability study. Sakarya Univ J Educ. 2017;7(2):385–97. doi: 10.19126/suje.296824

[53] Chen Z. Primary school teachers are immune: a journey in the sea of psychological well-being, buoyancy, and engagement. BMC Psychol. 2024;12(1):86. doi: 10.1186/s40359-024-01592-1 38383448 PMC10882816

[54] Dorji S, Sirasoonthorn P, Anusaksathien K. School Teachers in Rural Bhutan: Quality of Work Life, Well-Being and the Risks of Resignation. South Asia Research. 2019;39(3):270–84. doi: 10.1177/0262728019872038

[55] Zhang Z, Lee JC-K, Yin H, Yang X. Doubly latent multilevel analysis of the relationship among collective teacher efficacy, school support, and organizational commitment. Front Psychol. 2023;13:1042798. doi: 10.3389/fpsyg.2022.1042798 36687968 PMC9846613

[56] Dreer B. On the outcomes of teacher wellbeing: a systematic review of research. Front Psychol. 2023;14:1205179. doi: 10.3389/fpsyg.2023.1205179 37575417 PMC10421665

[57] White JMH. The critical role of happiness in education: Resilience, retention, and what happy teachers do differently [doctoral dissertation]. University of Northern Iowa. 2023.

[58] Bartlett JE, Kotrlik JW, Higgins CC. Organizational research: determining appropriate sample size in survey research. Inf Technol Learn Perform J. 2001;19(1):43–50.

[59] Faul F, Erdfelder E, Buchner A, Lang A-G. Statistical power analyses using G*Power 3.1: tests for correlation and regression analyses. Behav Res Methods. 2009;41(4):1149–60. doi: 10.3758/BRM.41.4.1149 19897823

[60] Arslan Y, Polat S. Örgütsel mutluluk ölçeğinin Türkçe’ye uyarlanması. Kuram Uygulamada Eğit Yönet Derg. 2017;23(4):603–22.

[61] Meyer JP, Allen NJ, Smith C. Commitment to organizations and occupations: extension and test of a three-component conceptualization. J Appl Psychol. 1993;78(4):538–51.

[62] Dağlı A, Elçiçek Z, Han B. Örgütsel Bağlılık Ölçeği’nin Türkçeye uyarlanması: Geçerlik ve güvenirlik çalışması. Elektron Sos Bilim Derg. 2018;17(68):1765–77. doi: 10.17755/esosder.445932

[63] Hair JF, Black WC, Babin BJ, Anderson RE. Multivariate Data Analysis. 7th ed. Harlow, UK: Pearson. 2014.

[64] Kline RB. Principles and Practice of Structural Equation Modeling. 4th ed. New York, NY, USA: Guilford Publications. 2016.

[65] Leech NL, Barrett KC, Morgan GA. SPSS for intermediate statistics: Use and interpretation. 2nd ed. New Jersey: Lawrence Erlbaum Associates. 2005.

[66] Büyüköztürk Ş. Sosyal bilimler için veri analizi el kitabı: İstatistik, araştırma deseni, SPSS uygulamaları ve yorum. Ankara: PegemA Yayıncılık. 2003.

[67] Field A. Discovering statistics using IBM SPSS Statistics. London: Sage Publications Limited. 2024.

[68] Hair JF, Hult GTM, Ringle CM, Sarstedt M. A Primer on Partial Least Squares Structural Equation Modeling (PLS-SEM). Thousand Oaks, CA, USA: SAGE; 2022.

[69] Braun V, Clarke V. Using thematic analysis in psychology. Qual Res Psychol. 2006;3(2):77–101. doi: 10.1191/1478088706qp063oa

[70] Miles MB, Huberman AM. Qualitative Data Analysis. Thousand Oaks, CA, USA: SAGE. 1994.

[71] Fornell C, Larcker DF. Evaluating structural equation models with unobservable variables and measurement error. J Mark Res. 1981;18(1):39–50. doi: 10.1177/002224378101800104

[72] Lam LW. Impact of competitiveness on salespeople’s commitment and performance. J Bus Res. 2012;65(9):1328–34. doi: 10.1016/j.jbusres.2011.10.026

[73] Henseler J, Ringle CM, Sarstedt M. A new criterion for assessing discriminant validity in variance-based structural equation modeling. J Acad Mark Sci. 2015;43(1):115–35. doi: 10.1007/s11747-014-0403-8

[74] Gold AH, Malhotra A, Segars AH. Knowledge management: An organizational capabilities perspective. J Manag Inf Syst. 2001;18(1):185–214. doi: 10.1080/07421222.2001.11045669

[75] Meyer JP, Allen NJ. A three-component conceptualization of organizational commitment. Hum Resour Manag Rev. 1991;1(1):61–89. doi: 10.1016/1053-4822(91)90011-Z

[76] Demircan T. Examining the relationship between teachers’ organizational commitment levels and organizational happiness. Uşak University. 2019.

[77] Ingsih K, Mursid A, Suhana S, Almadana AV, Ali S. Exploring the impact of corporate ethical values on organizational commitment: do trust in managers and employee well-being play a role?. J Glob Responsib. 2026;:1–16. doi: 10.1108/jgr-08-2023-0136

[78] Zhou S, Slemp GR, Vella-Brodrick DA. Factors associated with teacher wellbeing: a meta-analysis. Educ Psychol Rev. 2024;36(2). doi: 10.1007/s10648-024-09886-x

[79] Wright TA, Cropanzano R. Psychological well-being and job satisfaction as predictors of job performance. J Occup Health Psychol. 2000;5(1):84–94. doi: 10.1037//1076-8998.5.1.84 10658888

[80] McGuire D, McLaren L. The impact of physical environment on employee commitment in call centres: the mediating role of employee well-being. Team Perform Manag. 2009;15(1/2):35–48. doi: 10.1108/13527590910937702

[81] Thompson A, Bruk-Lee V. Employee Happiness: Why We Should Care. Applied Research Quality Life. 2020;16(4):1419–37. doi: 10.1007/s11482-019-09807-z

[82] Li M, Liu F, Yang C. Teachers’ Emotional Intelligence and Organizational Commitment: A Moderated Mediation Model of Teachers’ Psychological Well-Being and Principal Transformational Leadership. Behav Sci (Basel). 2024;14(4):345. doi: 10.3390/bs14040345 38667141 PMC11048059

[83] Xu Z, Pang NS-K. Promoting Teachers’ Organizational Commitment: The Effects of Authentic Leadership, Teachers’ Well-Being and Social-Emotional Competence. Behav Sci (Basel). 2024;14(10):862. doi: 10.3390/bs14100862 39457734 PMC11505086

[84] Özyıldırım G. Teachers’ occupational health: A structural model of work‐related stress, depressed mood at work, and organizational commitment. Psychol Sch. 2024;61(7):2930–48. doi: 10.1002/pits.23202

[85] Friedman IA. High- and low-burnout schools: School culture aspects of teacher burnout. J Educ Res. 1991;84(6):325–33.

[86] Gkliati A, Saiti A, Chletsos M, Bechlioulis AP. The relationship of minimum wage on employees’ well–being, job satisfaction and work–life balance. Int J Organ Anal. 2025. doi: 10.1108/ijoa-05-2024-4520

[87] Peñalver J, Salanova M, Martínez IM, Schaufeli WB. Happy-productive groups: How positive affect links to performance through social resources. J Posit Psychol. 2017;14:377–92. doi: 10.1080/17439760.2017.1402076

[88] Şahin H, Dursun A. Okul öncesi öğretmenlerinin iş doyumları: Burdur örneği. Mehmet Akif Ersoy Univ Eğit Fak Derg. 2009;9(18):160–74.

[89] Romero-Rodríguez LM, Castillo-Abdul B. Internal communication from a happiness management perspective: state-of-the-art and theoretical construction of a guide for its development. BMC Psychol. 2024;12(1):644. doi: 10.1186/s40359-024-02140-7 39522021 PMC11550556

[90] Bulut A. Examination of secondary school teachers’ perceptions of organizational happiness: A norm study [doctoral dissertation]. Gaziantep University; 2015.

[91] Argon T. Öğretmenlerin Sahip Oldukları Duygu Durumlarını Okul Yöneticilerinin Dikkate Alıp Almamalarına İlişkin Görüşleri. Abant İzzet Baysal Üniversitesi Eğitim Fakültesi Dergisi. 2015;15(1). doi: 10.17240/aibuefd.2015.15.1-5000128614

[92] Shao Y, Jiang W, Wang N, Zhang C, Zhang L. The impact of authentic leadership on the work engagement of primary and secondary school teachers: The serial mediation role of school climate and teacher efficacy. PLoS One. 2025;20(5):e0320839. doi: 10.1371/journal.pone.0320839 40408390 PMC12101708

[93] İnce M, Gül H. Yönetimde yeni bir paradigma: örgütsel bağlılık. Konya, Turkiye: Çizgi Kitabevi. 2005.

[94] Turan N. Bir Kamu Üniversitesi Personelinin Çalışma Mutluluğunu Etkileyen Faktörler: Nitel Araştırma Örneği. jecs. 2019;0(60):187–205. doi: 10.26650/jecs2019-0011

[95] Harter J, Adkins A. What great managers do to engage employees. Harv Bus Rev. 2015 Apr 02. Available from: https://hbr.org/2015/04/what-great-managers-do-to-engage-employees

[96] Moçoşoğlu B, Kaya A. Okul yöneticileri ve öğretmenlerin örgütsel sessizlik ile örgütsel mutluluk düzeyleri arasındaki ilişki: Şanlıurfa ili örneği. Harran Maarif Derg. 2018;3(1):52–70. doi: 10.22596/2018.0301.52.70

[97] Gavin J, Mason R. The virtuous organization: The value of happiness in the workplace. Organ Dyn. 2004;33(4):379–92. doi: 10.1016/j.orgdyn.2004.09.005

